# Deformable Catalytic Material Derived from Mechanical Flexibility for Hydrogen Evolution Reaction

**DOI:** 10.1007/s40820-023-01251-x

**Published:** 2023-11-24

**Authors:** Fengshun Wang, Lingbin Xie, Ning Sun, Ting Zhi, Mengyang Zhang, Yang Liu, Zhongzhong Luo, Lanhua Yi, Qiang Zhao, Longlu Wang

**Affiliations:** 1 College of Electronic and Optical Engineering & College of Flexible Electronics (Future Technology), Nanjing University of Posts & Telecommunications (NJUPT), 9 Wenyuan, Nanjing, 210023 People’s Republic of China; 2 Key Laboratory of Environmentally Friendly Chemistry and Applications of Ministry of Education, School of Chemistry, Xiangtan University, Xiangtan, 411105 People’s Republic of China; 3 State Key Laboratory of Organic Electronics and Information Displays & Jiangsu Key Laboratory for Biosensors, Institute of Advanced Materials (IAM) & Institute of Flexible Electronics (Future Technology), Nanjing University of Posts & Telecommunications, 9 Wenyuan, Nanjing, 210023 People’s Republic of China

**Keywords:** Deformable catalytic material, Micro–nanostructures evolution, Mechanical flexibility, Hydrogen evolution reaction

## Abstract

Deformable catalytic material with excellent flexible structure is a new type of catalyst that has been applied in various chemical reactions, especially electrocatalytic hydrogen evolution reaction (HER). In recent years, deformable catalysts for HER have made great progress and would become a research hotspot. The catalytic activities of deformable catalysts could be adjustable by the strain engineering and surface reconfiguration. The surface curvature of flexible catalytic materials is closely related to the electrocatalytic HER properties. Here, firstly, we systematically summarized self-adaptive catalytic performance of deformable catalysts and various micro–nanostructures evolution in catalytic HER process. Secondly, a series of strategies to design highly active catalysts based on the mechanical flexibility of low-dimensional nanomaterials were summarized. Last but not least, we presented the challenges and prospects of the study of flexible and deformable micro–nanostructures of electrocatalysts, which would further deepen the understanding of catalytic mechanisms of deformable HER catalyst.

## Highlights

- The main effects of deformation of flexible catalytic materials on the catalytic hydrogen evolution reaction performance are discussed, and a series of novel strategies to design highly active catalysts based on the mechanical flexibility of low-dimensional nanomaterials are summarized in detail.
- This review provides a strategic choice for the rational design of low-cost and high-performance industrialized electrocatalysts.

## Graphical abstract

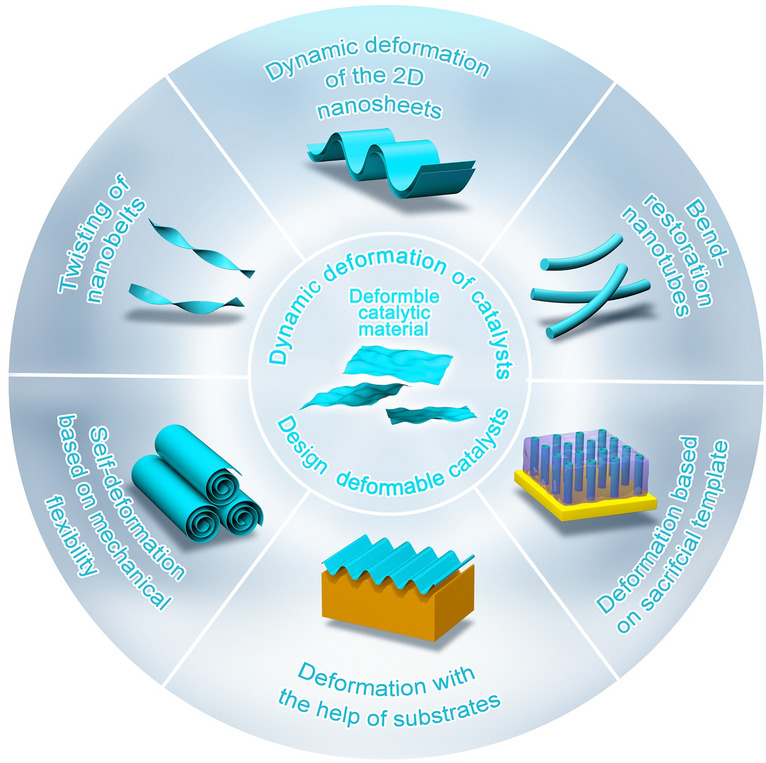

## Introduction

Hydrogen evolution reaction (HER) is the core component in water splitting devices and seriously limits energy efficiency due to sluggish reaction kinetics [1–4]. The electrocatalytic HER performance is mainly determined by the catalyst activity [5–7]. Low-dimensional catalysts have become mainstream due to their adjustable and flexible structures [8–10]. The HER catalytic performance of low-dimensional catalysts always evolves dynamically, originating from the variable structures based on excellent mechanical flexibility. On the one hand, the tension and shaking force generated by the escape and break of surface hydrogen bubbles under the stimulation of high currents in the HER catalytic process could lead to structural deformation and then further affect catalytic performance [11–16]. In situ exploration of the structure–activity relationship between the deformable structure and the catalytic performance during the catalytic reaction is very necessary [17–19]. On the other hand, it is also a very sensible strategy to design high active catalysts based on the mechanical flexibility and deformation properties [20–22].

It has been widely reported that the morphologies of low-dimensional catalysts are prone to change during the HER process [23–32]. Structural deformation of the catalyst would occur under the action of the flowing fluid and bubbles, which has an impact on HER catalytic performance. For example, Wang et al. [33] demonstrated that the shear force induced by flowing fluid in HER process could make the catalyst deform, then improve the pristine catalytic activity and enhance the mass transform. The impact and drag of hydrogen bubble breaking and separating on the catalyst would break rigid catalytic materials and cause HER performance degradation. Zhai et al. [34] indicated that mechanical flexible nanotube could alleviate the influence of the tension and shaking force generated by bubbles. In fact, the flexibility and variability of low-dimensional nanomaterials can also be used to design highly active catalysts. Chen et al. [35] designed MoS_2_/WS_2_ nanoscrolls with tube-wrapped structure by rolling and curling heterojunction bilayer membranes. The collective effect of local strain and the layer-by-layer wrapped structure could facilitate the electron transfer process to speed up HER reaction rate. Our group [36] successfully synthesized moiré superlattices (MSLs) based on the initial mechanical flexibility of WS_2_ nanobelts. The ultrathin WS_2_ nanobelts with high flexibility spontaneously bend and twist to form helix nanocones, resulting in formation of MSLs induced by the S–W–S layers twisting. The WS_2_ MSLs delivered excellent HER performance based on special physical and chemical properties of MSLs. Deformation modulation provides a new dimension for catalytic material property modulation. Wang et al. [37] considered that surface curvature plays an important role in electrocatalysis, but it is still necessary to summarize the latest progress of the deformation of catalytic materials in improving HER properties and designing deformed catalysts with high activity.

In this paper, we reviewed the deformable catalytic material derived from mechanical flexibility for HER. On the one hand, we focus on the main effects of deformation of flexible catalytic materials on the catalytic HER performance, such as the increase of mass transfer rate, optimization of catalyst active sites and enhancement of catalytic stability during reaction process. On the other hand, we summarize a series of novel strategies to design highly active catalysts based on the mechanical flexibility of low-dimensional nanomaterials [38, 39]. Finally, we present the challenges and prospects of flexible deformable micro–nanoelectrocatalyst research and provide our insights.

## Characterize of Deformable Catalytic Materials

The characterization of deformable materials is crucial for the design of deformable catalytic materials and the study of their morphology changes during the catalytic process. High-resolution characterization instruments provide a broad arsenal for clear identification of catalytic sites and in-depth exploration of catalytic mechanisms [40]. These characterization techniques can be classified into direct imaging and indirect spectroscopy. In situ high-resolution imaging techniques are powerful for tracking the surface morphology and bulk phase evolution of catalysts in a rather limited area. In situ SEM is the most intuitive approach to visualizing surface morphological changes and even local structural evolution around atomic sites. By means of in situ SEM technology, the morphology changes of flexible catalytic materials during the catalytic process can be fully understand [41]. However, such characterization tools to investigate the deformation of catalytic materials are still lacking, and we have proposed the need to develop such in-situ HRTEM techniques in the prospect section.

Regarding diverse spectroscopic techniques, they specialize in in-depth analysis of element classes, chemical microenvironments, and active site coordination structures, which are indispensable indexes for studying how deformation of catalytic materials affects catalytic activity. Benefiting from its low sample preparation requirement, facile operation, non-contacting mode and quick detection, Raman spectroscopy can be used to record the real-time evolution of deformed catalyst surface, local coordination environment and intermediate species during the reaction process. X-ray diffraction (XRD) is used to study the structural changes and surface macroscopic stresses of the deformed catalyst. Excellent in collecting the compositional and structural information on the shallow region < 2 nm below the sample surface, XPS is suitable for analyzing the change of element valence state of the deformed catalytic materials. X-ray absorption spectroscopy (XAS) provides comprehensive information about the chemical environment and the electronic structure of the detected atoms. By analyzing the X-ray changes before and after the incident, information about the chemical environment surrounding the constituent atoms will be presented. Similarly, we propose in our outlook the need to develop advanced spectral techniques to further enhance characterization [42].

## Dynamic Deformation of Catalysts in the HER Process

The tension and shaking force generated from the escaping and breaking of surface bubbles have a severe impact on materials, resulting in the deformation of low-dimensional catalyst under high current density in the HER process [43]. The difference in deformation is caused by the material's own morphology and the surrounding environment. In this section, we will discuss the law of low-dimensional catalyst deformation such as geometry-induced variable effects, expansion and oscillation of stacked three-dimensional nanosheet superstructure, flexible twisting of nanobelts and bending of nanotubes, as well as their effects on the catalytic activity in the real reaction process [44–47]. The morphology evolution of catalyst during the reaction process would not only effectively improve the electrocatalytic activity, but also promote mass transfer as well as bubble dynamics.

### Dynamic Deformation of the 2D Nanosheets

The ultrathin nanosheets would spontaneously deform due to their flexible characteristics during the catalytic process [48, 49]. The deformation of ultrathin nanosheets could significantly enhance mass transport during the electrocatalytic process and then improve the catalytic activity [50–56].

**Fig. 1:**
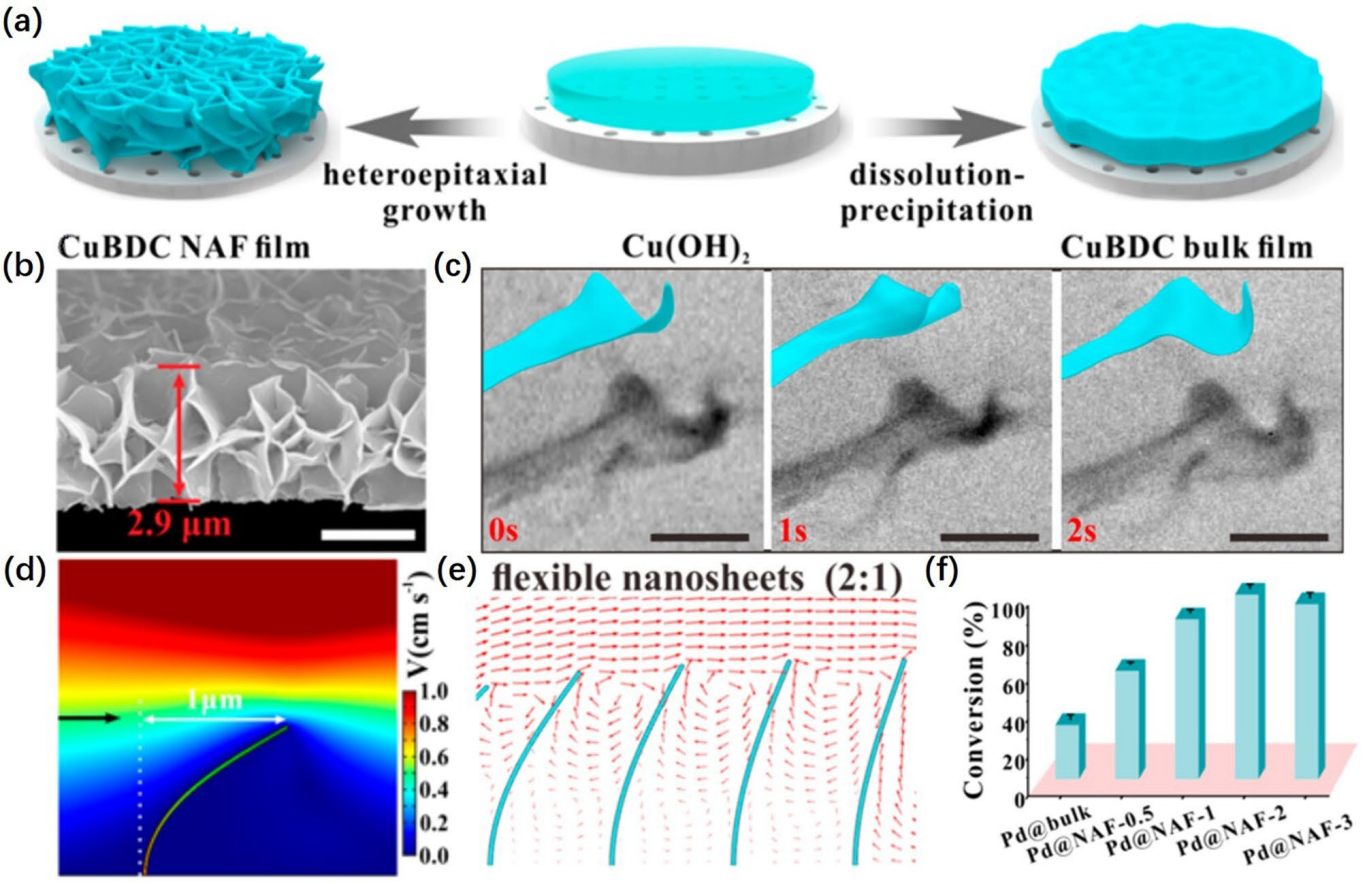
**a** Schematic overview of the preparation of the MOF NAF film and the bulk-type film.** b** Cross-sectional scanning electron microscope (SEM) images of the as-synthesized MOF NAF products. **c** In situ TEM was used to observe the dynamic deformation of the nanosheets at the edge of the NAF film in the fluid. **d** Flow field around the single MOF nanosheet, where in the arrow indicates the flow direction. The deformation of the nanosheet (*E* = 0.2 GPa) was 1.0 μm at 1.0 cm s^−1^ velocity. **e** Velocity vectors evolving with time. The numbers in brackets represent the ratio between the height and spacing of the nanosheets. **f** Hydrogenation conversions for various alkenes catalyzed by the Pd NCs@MOF composites [33], © American Chemical Society 2020

The dynamic state near the catalyst surface can have an impact on the rate of product attachment and reactant detachment. The deformation of flexible catalysts under eddy currents and perturbations has been demonstrated to be effective in enhancing the mass transfer at the catalyst surface. Wang et al. [33] prepared Pd nanocube (NC) catalyst encased in a soft MOF nanosheet assembly framework (NAF) by heteroepitaxy growth (Pd NCs@MOF). The local mass transport was facilitated by fine-tuning the deformation of MOF NAFs. As shown in Fig. 1a, the NAF thin films assembled by deformable nanosheets were prepared by heteroepitaxial growth method. The deformable nanosheets were demonstrated in scanning electron microscope (SEM) image accessed in Fig. 1b. As illustrated in Fig. 1c, the geometry-induced variable 2D-nanosheets with mechanical flexible characteristics that showed a distorted shape to adapt to the fluid. The nanosheets underwent a deformation of 1 μm when subjected to a flow rate of ~ 1 cm^−1^ s^−1^ (Fig. 1d). This dynamic deformation of the nanosheets in the fluidic system greatly disturbs the surface of the nanosheets and facilitates the mass transfer near the solid boundary. As shown in Fig. 1e, the swaying of the flexible nanosheets produced strong vortices effect, which could significantly enhance the transport of reactants, thus improving the reaction rate. The catalytic hydrogenation of alkenes performance of Pd NCs@MOF composite is shown in Fig. 1f. The Pd NCs @ MOF NAF-2 (thickness of 2.9 μm) showed the highest catalytic efficiency. Deformable nanosheets driven by the shear force of flowing fluid accelerate the interaction between reactants and catalysts and effectively regulate HER reaction rate during HER process, which has a reference for using deformable catalytic materials to regulate HER properties of nano-catalysts.

### Twisting of Nanobelts

Mechanical flexibility can lead to uneven stress in the nanobelts, which lead to the formation of uniform nanohelices due to a rigid lattice rotation or twisting during the HER process [57]. The strain and phase transition accompanying the distortion are introduced into the nanobelts to realize the regulation of catalytic performance [30, 31, 58].

**Fig. 2:**
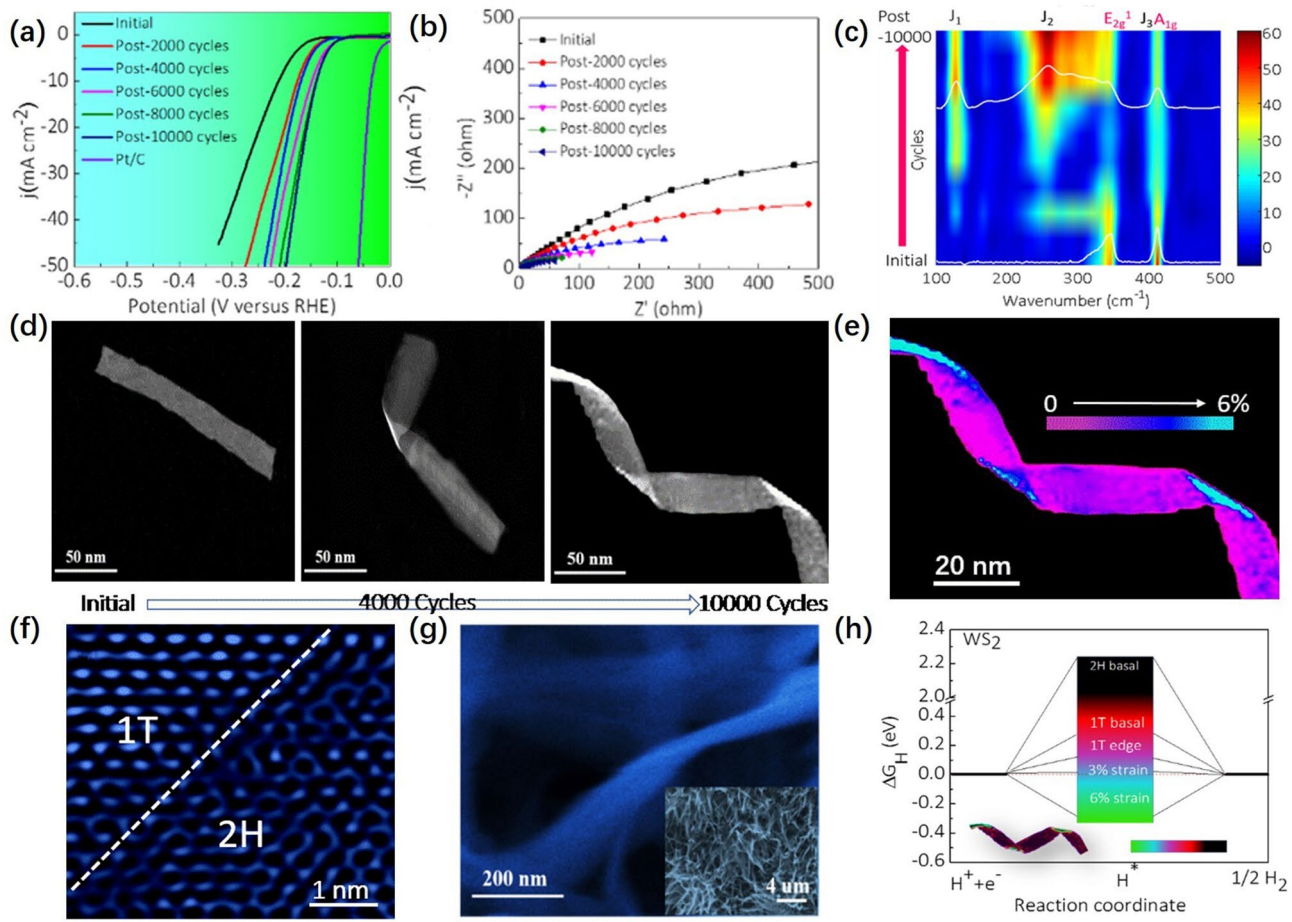
**a** Electrochemical data of WS_2_ nanobelts in H_2_SO_4_ (0.5 M). Polarization curves are shown as a function of number of potential cycles and compared to that of the Pt benchmark catalyst. **b** Nyquist plots with the number of potential cycles. **c** In situ Raman spectroscopy as a function of CV cycles (phase transformation process). **d** Morphological evolution of WS_2_ nanobelts with the increasing number of cycles. **e** TEM image with false colors of a WS_2_ nanohelices after cycling. **f** A HRTEM image of a WS_2_ nanohelices showing its in-plane 1T–2H heterostructure. **g** SEM image of WS_2_ nanohelices (after WS_2_ nanobelts were cycled for 20,000 times). **h** Comparison of the influence of different sites and strains for the 1T and 2H-WS_2_ phases on the HER performance. *ΔG*_H_^*^ diagram of the different H adsorption sites. The inset is a TEM image (with false color) of WS_2_ nanohelices [59], © Elsevier B.V. 2019

Nanobelts, as a typical two-dimensional TMDs material, have attracted much attention for their unique low-dimensional properties. The phase transition from 2H to the active 1T phase could easily occur through the sliding of S-atom planes within the layer. Our team [59] prepared high active 1T-WS_2_ nanohelices by in situ topological transformation of 2H-WS_2_ nanobelts. As shown in Fig. 2a, the HER catalytic activity of the WS_2_ nanobelts after CV cycling keeps approaching that of the noble metal Pt compared to the pristine WS_2_ nanobelts. The charge transfer resistance decreased significantly with the increasing the number of CV cycles in Fig. 2b, indicating that the WS_2_ nanobelts after CV cycles not only have higher catalytic activity but also have high charge transfer rate. In situ Raman spectroscopy in Fig. 2c showed the phase transition of WS_2_ nanobelts from 2H to 1T, and the degree of phase transition increased with the increase of CV cycles.

As shown in Fig. 2d, the morphology of WS_2_ nanobelts gradually twisted or distorted with the increasing number of CV cycles during HER and 0–6% strain was systematically introduced to the surface of the WS_2_ nanohelices after cycling (Fig. 2e). High Resolution Transmission Electron Microscope (HRTEM) images of the WS_2_ nanohelices in Fig. 2f implied the coexistence of 1T and 2H phases. The SEM images of WS_2_ nanobelts after 20,000 potential cycles in 0.5 M H_2_SO_4_ solution from 0.2 to − 0.2 V (relative to RHE) in Fig. 2g showed that the distorted WS_2_ nanohelices interconnect with each other and form an open porous framework, which not only exposes the active edge, but also allows easy access of the reagent to the inner surface of the 1T-WS_2_ electrode, providing a good reaction environment. As illustrated in Fig. 2h, the atomic hydrogen adsorption free energy of distorted 1T-WS_2_ monolayers with different strains was calculated by DFT. The introduction of strain could significantly affect the *ΔG*_H_^*^ on the surface of 1T-WS_2_, and *ΔG*_H_^*^ approaches 0 at a strain of 3.0%. This self-optimizing behavior has practical advantages over other more complex methods for optimizing WS_2_-based catalysts, as it enables a highly scalable treatment with minimal additional processing, resulting in a better catalytic performance.

### Bend-restoration Nanotubes

The tension and shaking force generated during bubble escape and break are widely considered to be factors for the poor stability of electrocatalytic processes, especially at high current densities. Deformation of flexible materials effectively relieve damaging stresses from the surrounding environment [60–62].

**Fig. 3:**
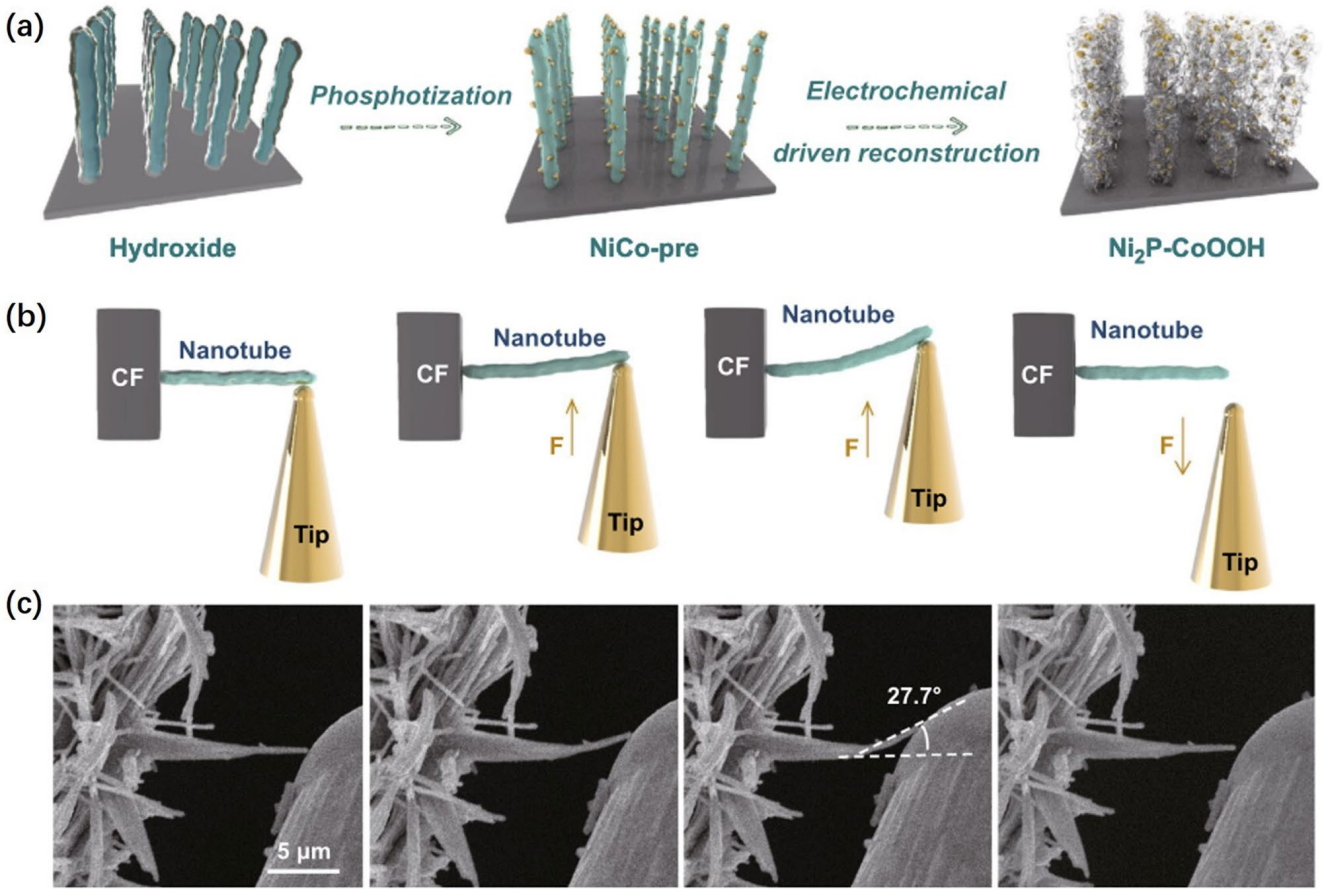
**a** Synthesis process of two-dimensional sheet-encapsulated tubular array catalysts. **b**, **c** In situ bending deformation and restoration measurement by SEM probe [34], © Springer Nature 2020

The rapid generation of bubbles during electrocatalytic HER reaction at high current densities requires catalysts that can withstand repeated deformation. Therefore, suitable mechanical properties are required for high-current HER catalysts. Zhang et al. [34] prepared the 2D CoOOH sheet-encapsulated Ni_2_P into tubular arrays electrocatalytic system (Fig. 3a). In situ bending deformation and restoration measurement indicated that the high mechanical toughness of nanotubes could buffer the shock of electrolyte convection, hydrogen bubble rupture, and evolution through the release of stress, insuring the long cycle stability (Fig. 3b). As shown in Fig. 3c, the maximum bending angle of the single nanotube could withstand is up to 27.7°. When the external force is removed, the nanotube could recover intact, showing excellent mechanical properties. Therefore, the Ni_2_P–CoOOH nanotubes with high impact strength, high torsional strength and high fatigue strength were beneficial to achieve high efficiency HER [63].

## Designing Highly Active Catalysts Based on Mechanical Flexibility

Based on the mechanical flexibility of nanomaterials, highly active catalysts with specific morphology can be further constructed [64, 65]. There are a number of different design strategies, mainly divided into three. Such as self-deformation based mechanical flexibility, with the help of curved substrates and flexible deformable substrate, sacrifice template strategy. Each of these methods has its own advantages and disadvantages. The self-deformation based on mechanical flexibility is simple and easy to operate, but it is uncontrollable. The strategy with the help of substrate has a strong ability to control the deformation, but the dependence and requirement on the substates are very strict. The preparation strategy based on sacrificial template can accurately control the degree of deformation, but it needs to find the right sacrificial agent.

### Self-Deformation based on Mechanical Flexibility

The strain introduced on the surface of the nanoscrolls can optimize the HER performance of the catalyst. Based on the self-mechanical flexibility of nanomaterials, nanofilms or nanosheets can be assembled by drying, solvent and freezing into nanoscrolls with bending strain on the surface. In addition, the planar catalytic materials can also be rolled to form nanoscrolls structures through defect engineering [66, 67].

**Fig. 4:**
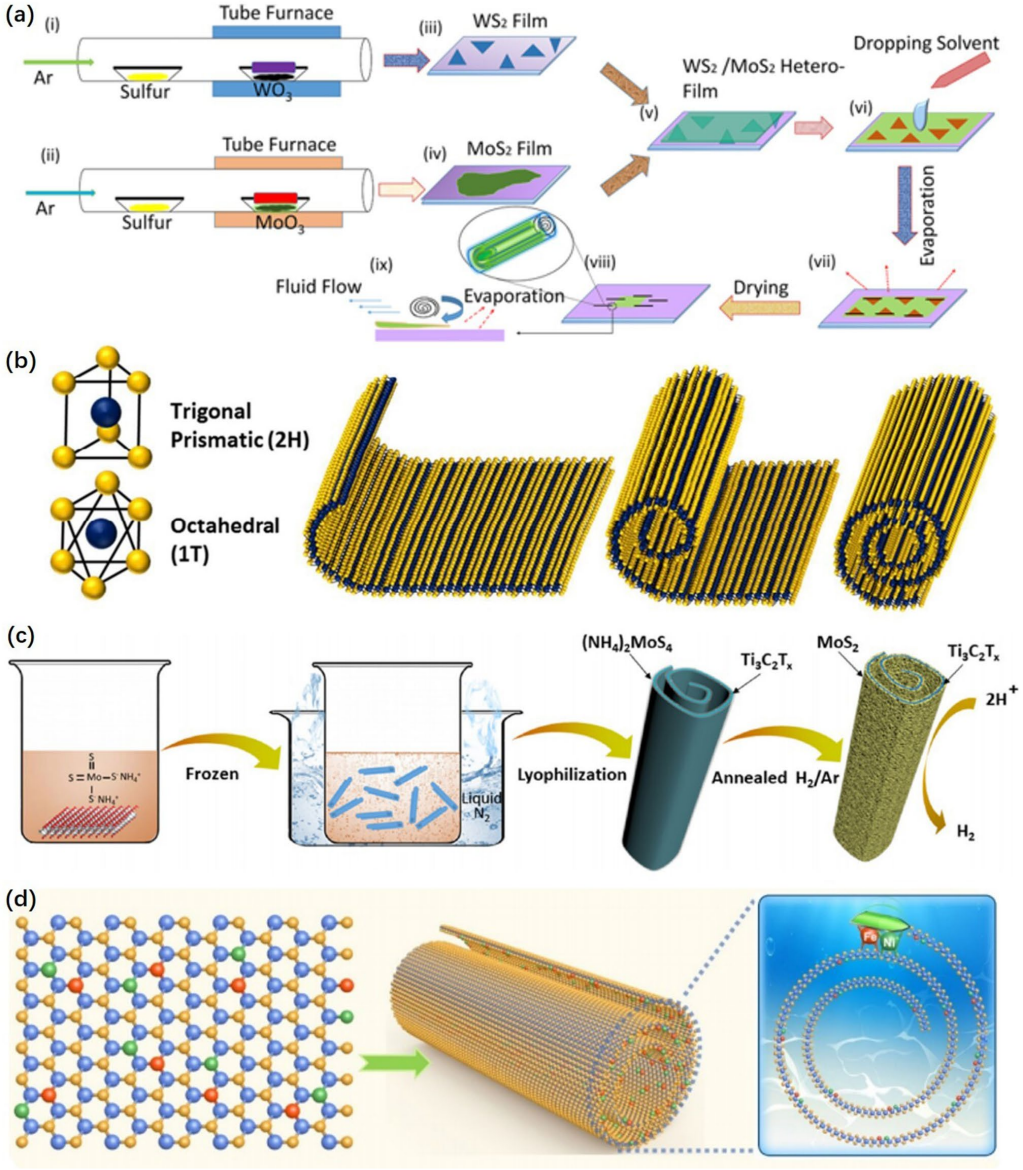
**a** Schematic diagram of WS_2_/MoS_2_ heterojunction nanoscroll (NS) fabrication in different steps: monolayer (i) WS_2_ and (ii) MoS_2_ growth on Si/SiO_2_ substrate via chemical vapor deposition (CVD). A layer of PMMA is spin-coated on both (iii) WS_2_ and (iv) MoS_2_ films. (v) MoS_2_ is transferred onto the WS_2_ film through a wet transfer technique. (vi) After acetone washing, the evaporating solvent is cast onto the heterojunction film. (vii) The solvent is evaporated after a specific time, and liquid is intercalated between the film and the substrate. (viii) After drying for several minutes, the heterofilm is converted into heterojunction-NS (zoomed-in image: schematic of a single heterojunction-NS) [35], © American Chemical Society 2022. **b** 2H MoS_2_ sheets and 1T@2H MoS_2_ scrolls [68], © Royal Society of Chemistry 2018. **c** Preparation of the “nanoroll” like MoS_2_/Ti_3_C_2_T_*x*_ hybrid by combining liquid nitrogen-freezing and subsequent annealing [71], © Elsevier B.V. 2018. **d** The DAs in two adjacent layers are assembled atomically into nanocoils within the two-dimensional limit [72], © John Wiley & Sons, Inc 2023

Chen et al. [35] combined MoS_2_ and WS_2_ into hetero-film. Then nanoscrolls (NS) were formed through evaporation assembly based on hetero-film mechanical flexibility and wake interaction with the substrate (Fig. 4a). Suh Dong Hack et al. [68] added a self-assembled material which was used for rolling up two dimensional materials on the exfoliated 2H-MoX_2_ (X; S, Se and Te) nanosheets. As shown in Fig. 4b, the free MoX_2_ sheets were rolled upward into a scroll-like structure to form 1T-MoX_2_ nanoscrolls based on the mechanical flexibility of the MoX_2_ nanosheets and the introduced bending strain. On this basis, the research group also used MoS_2_ sheets decorated with noble nanoparticles to prepare nanoscrolls with a high bending strain [69, 70].

Fan et al. [71] formed Ti_3_C_2_T_*X*_ nanoscrolls based on the self-mechanical flexibility of the exfoliated Ti_3_C_2_T_*X*_ nanosheets combined with liquid nitrogen freezing shrinkage. After annealing under H_2_/Ar atmosphere, MoS_2_ crystals with vertical alignment Ti_3_C_2_T_*x*_ were formed in situ on the surface of Ti_3_C_2_T_*X*_ nanoscrolls (Fig. 4c). As illustrated in Fig. 4d, Yuan et al. [72] anchored Ni and Fe dual atoms (DAs) on S vacancies of 2D MoS_2_ planar films with mechanical flexibility and formed NiFe@MoS_2_ nanoscrolls by self-curving treatment. Compared with conventional planar catalysts, nanoscrolls catalysts could introduce controlled bending strain to improve the catalytic activity of catalysts effectively.

Vertically stacked 2D materials with small azimuth deviations or lattice mismatches produce unique global structural periodicity and symmetry which was as the moiré superlattice (MSL) [73–75]. Based on the flexible mechanism of 2D material, it can generate the interlayer distortion to form MSL by crimping deformation.

**Fig. 5:**
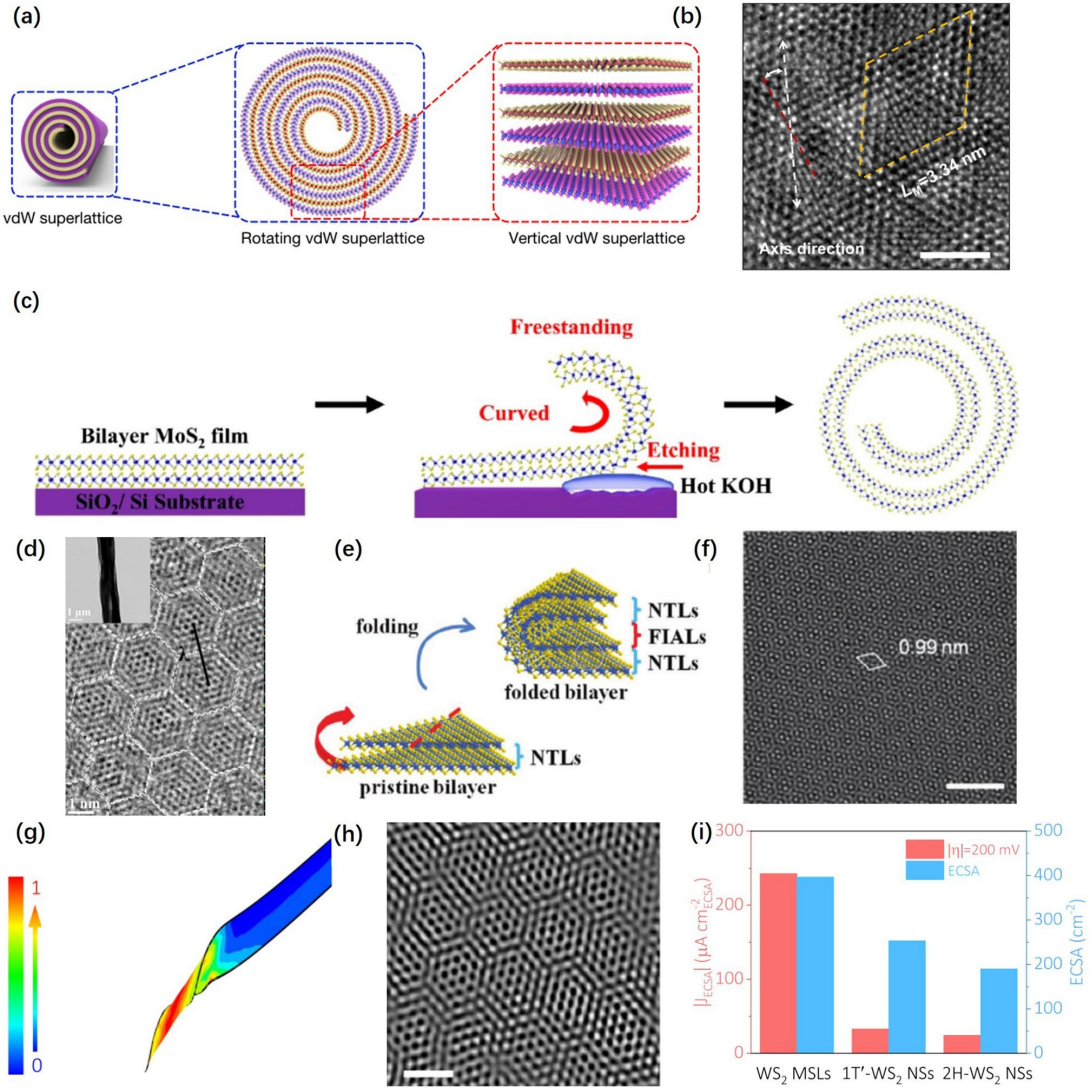
**a** Enlarged schematic of a vdW heterostructure volume and a schematic of a higher-order vdW superlattice.** b** High-angle annular dark-field STEM image of the periodic moiré superlattices of the SnS_2_/WSe_2_ vdW superlattice roll-up; the yellow dashed rhombus is the corresponding moiré unit cells with moiré superlattice constant *L*_M_ = 3.34 nm. Scale bar, 2 nm [76], © Springer Nature 2021. **c** Schematic diagram of the forming process of the MoS_2_ nanoscroll. **d** HRTEM image of the MoS_2_ nanoscroll. The inset is a low-magnification TEM image of the MoS_2_ nanoscroll [77], © American Chemical Society 2019. **e** Fabrication of one-interface-twisted multilayer MoS_2_ via a paraffin-assisted folding strategy. **f** The MSLs structures in TEM images. White rhombus, unit cells for moiré patterns. Experimentally measured period is labeled by the side. Scale bar, 3 nm [78], © Wiley–VCH GmbH 2022. **g** Finite element calculation of twisted nanobelt strain. The color bar shows the relative scale of the strain distribution. **h** Enlarged HRTEM characterization. Scale bar, 1 nm. **i** Comparison of the ECSA and J_ECSA_ (at − 0.2 V vs. RHE) of WS_2_ MSLs, 1T’-WS_2_ NSs, and 2H-WS_2_ NSs [36], © Springer Nature 2023

The preparation of MSLs by deformation of mechanically flexible materials is an effective physical method to precisely control the interlayer distortion. Duan et al. [76] prepared MSLs by exploiting the capillary effect and triggering the natural curling of vertical heterojunctions (Fig. 5a). The MSLs were demonstrated in image of single hole in a high-angle annular dark field STEM (HAADF-STEM) image accessed in Fig. 5b. Yuan et al. [77] introduced tensile strain to flexible MoS_2_ films to achieve the transition from planar MoS_2_ films to twisted MoS_2_ nanoscrolls with MSLs (Fig. 5c). HRTEM image of the MoS_2_ nanoscroll in Fig. 5d presented a scroll-like morphology and confirmed the presence of MSLs. Based on the mechanical flexibility of multilayer MoS_2_, Liu et al. [78] successfully prepared a multilayer MoS_2_ MSLs structure with only one layer interface for twisted stacking by a simple paraffin-assisted folding process of non-twisted stacked multilayer MoS_2_ (Fig. 5e). The TEM image in Fig. 5f proved that the method can be effectively prepared MSLs. Our research group [36] synthesized locally twisted spiral WS_2_ MSLs derived from mechanical flexibility by solvothermal method. The WS_2_ nanobelts would transfer into WS_2_ nanocones under the unbalanced forces. Finite element calculation of twisted nanobelt strain in Fig. 5g indicated that the strain was locked in the end of the WS_2_ nanocones. The deformation would further trigger the twisting of layers to form MSLs which could be demonstrated by HRTEM as shown in Fig. 5h. The electrocatalytic activity was evaluated by measuring the electrochemically active surface area (ECSA). As shown in Fig. 5i, the ECSA value of WS_2_ MSLs (396.6 cm^2^ ECSA) is much higher than that of 1T'-WS_2_ NSs (253.3 cm^2^ ECSA) and 2H-WS_2_ NSs (190.0 cm^2^ ECSA). The results show that the WS_2_ MSLs has more abundant active sites for electrochemical hydrogen evolution. The WS_2_ MSLs showed better HER performance compared with representative non-precious metal HER electrocatalysts reported in recent years. The MSLs with high HER activity derived from mechanical flexibility would become a very promising catalyst.

Core-shells with high nanoscale curved and multilayer structure have high structural stability, high conductivity, large specific surface area, and suitability for electrocatalysis. Nanosheets could be wrapped on a spherical core by annealing or heteroepitaxial growth to form this special structure and have a controllable number of layers [79–84].

**Fig. 6:**
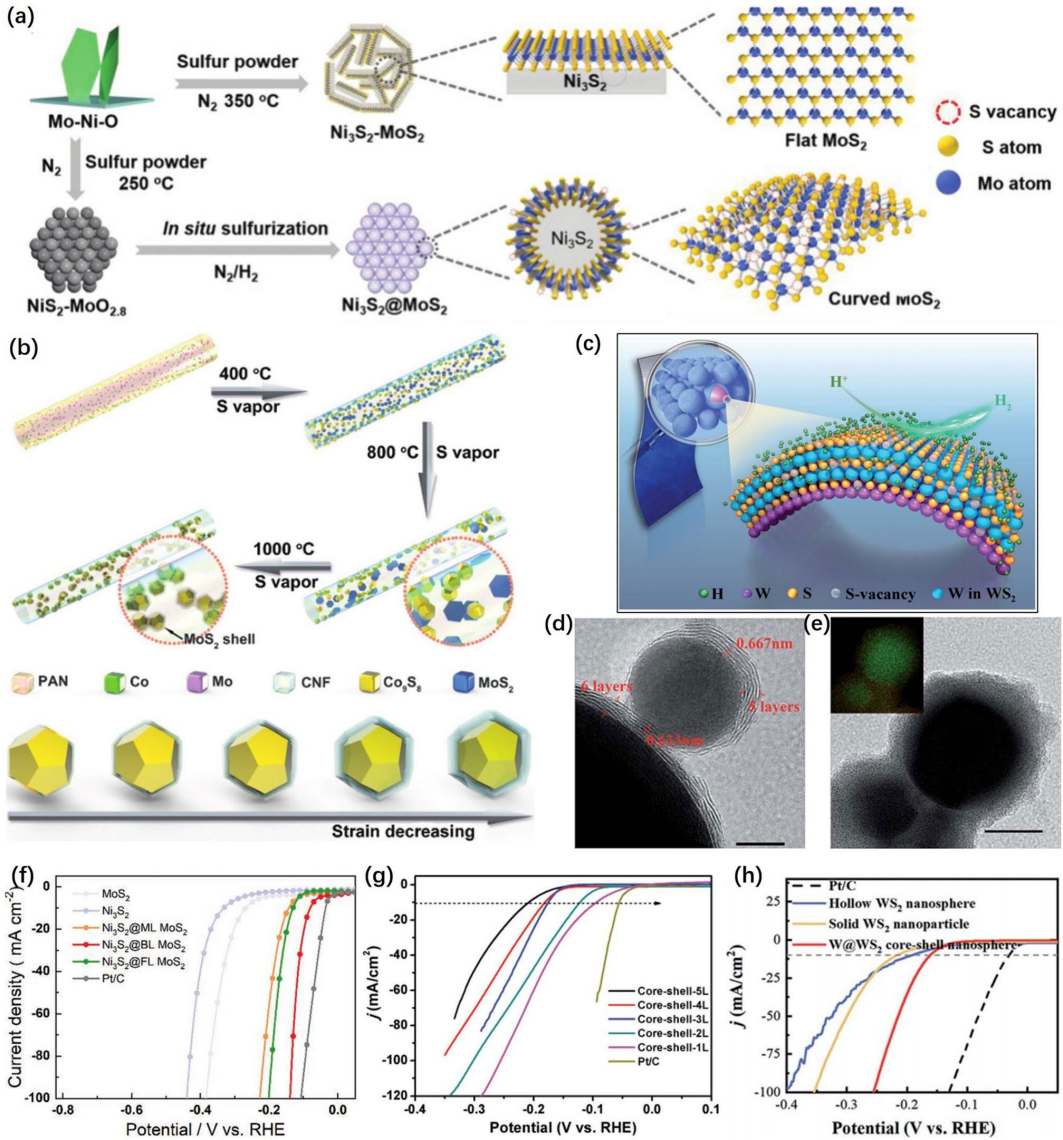
**a** Schematic of the synthetic process of Ni_3_S_2_–MoS_2_ (unstrained) and Ni_3_S_2_@MoS_2_ (biaxially strained) heterostructures [85], © Wiley–VCH GmbH 2022. **b** Synthetic process for Co_9_S_8_/MoS_2_ core/shell nanocrystals with precisely controlled shell numbers supported on CNFs. [86], © WILEY–VCH Verlag GmbH & Co. KGaA, Weinheim 2018. **c** Schematic illustration of the multifunctional W@WS_2_ CSNSs. **d** HRTEM and the corresponding EDS mapping images of the exfoliated nanospheres.** e** HRTEM images of the hierarchically curved W–S nanosheets [87], © The Royal Society of Chemistry 2021. **f** HER polarization curves of CFC, Ni_3_S_2_, Ni_3_S_2_@ML MoS_2_, Ni_3_S_2_@BL MoS_2_, Ni_3_S_2_@FL MoS_2_, and Pt/C [85], © Wiley–VCH GmbH 2022. **g** Polarization curves of Co_9_S_8_/nL MoS_2_ (*n* = 1–5) core/shell nanocrystals supported on CNFs and commercial Pt/C catalyst in 0.5 M H_2_SO_4_. Scan rate: 2 mV s^−1^ [86], © WILEY–VCH Verlag GmbH & Co. KGaA, Weinheim 2018. **h** LSV curves (iR-corrected) with a scan rate of 5 mV s^−1^ [87], © The Royal Society of Chemistry 2021

A novel in situ self-vulcanization strategy as shown in Fig. 6a was developed by Fan et al. [85] to achieve a biaxially strained single crystal Ni_3_S_2_@MoS_2_ core–shell nanostructure with adjustable layers, which optimized the active site of catalyst and enhanced H adsorption on catalyst surface. It showed outstanding catalytic activity of HER. Guo et al. [86] prepared Co_9_S_8_/MoS_2_ core/shell nanocrystals (Co_9_S_8_/MoS_2_-CNFs) supported on carbon nanofibers by a vapor-assisted method (Fig. 6b), in which two-dimensional 2H-MoS_2_ with a precise controllable layer was loaded on the Co_9_S_8_ nanocrystals. The catalytic performance was improved by precisely controlling biaxial strain in Co_9_S_8_/M_O_S_2_ core/shell nanocrystals.

Shen et al. [87] successfully prepared tungsten (W)@tungsten disulfide (WS_2_) core–shell nanospheres (CSNSs) with biaxial strain. As shown in Fig. 6c, the highly curved WS_2_ nanosheets on the W core promote the generation of defect sites. The HRTEM images in Fig. 6d showed that single nanosphere has an obvious core–shell structure. Figure 6e shows that the shell of CSNSs is composed of six layers of WS_2_ nanosheets grown from crystalline W cores. The high strain on the surface of core–shell structure could improve the intrinsic activity of exposed active sites. In fact, the catalysts performance has been greatly improved based on the biaxial strain method (Fig. 6f–h). Controlling the shell numbers of core–shell structures could precisely adjust the biaxial strain introduced into the catalyst surface to optimize the active site effectively. Therefore, the core–shell structure catalyst still has a very good development space [88].

### Deformation with the Help of Substrates

#### Curved Substrates

The 2D nanosheets grown on curved substrates are deformed by mutual extrusion due to their flexible characteristics and the curved effect of the substrate. This structure could maximize the exposure of active sites to enhance HER activity. The HER activity of nanosheets could be further improved by stripping the multilayer nanosheets on the substrate into single-layer or few-layer nanosheets [89–91].

**Fig. 7:**
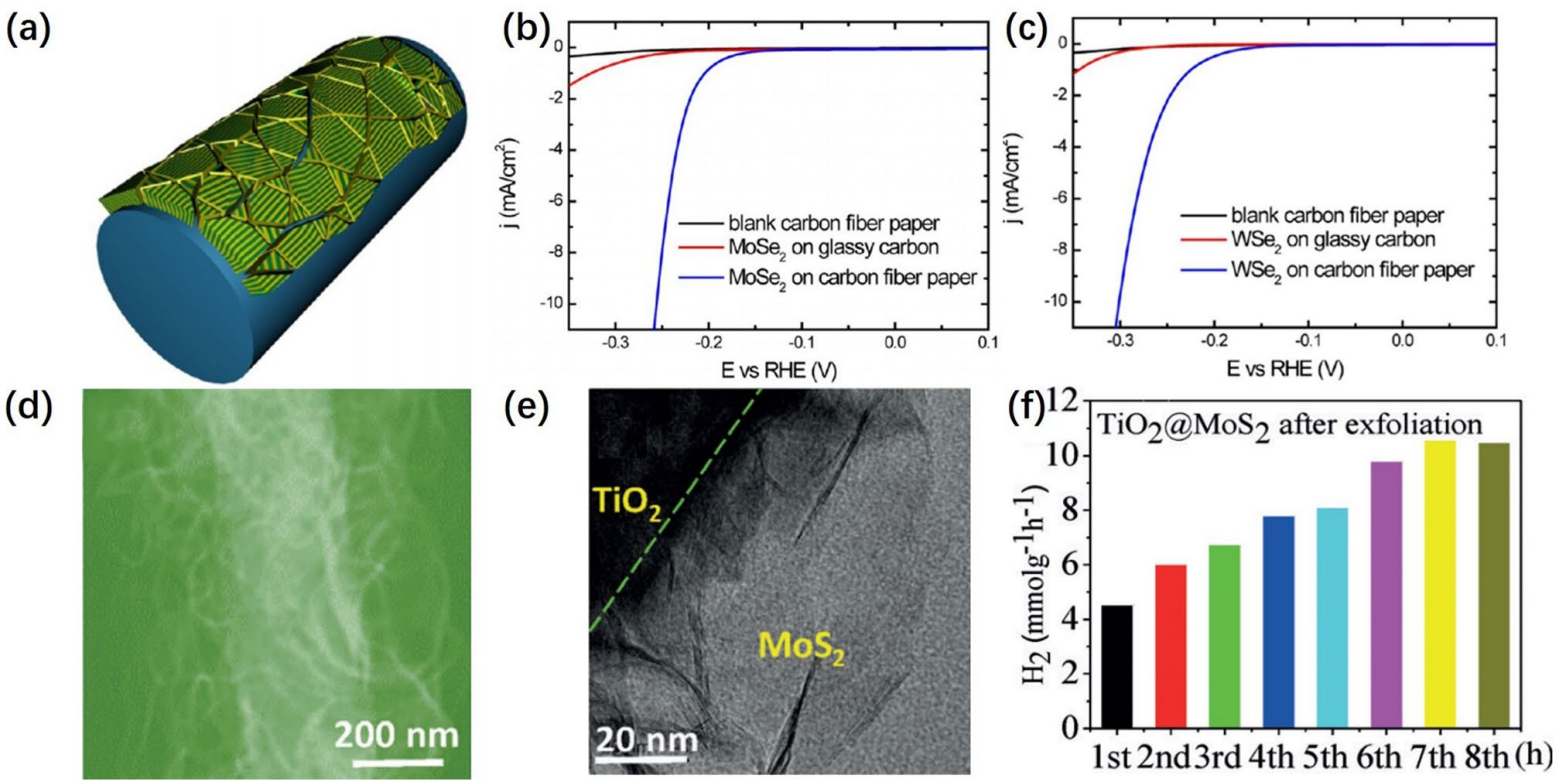
**a** Molybdenum (or tungsten) dichalcogenide nanofilm with molecular layers perpendicular to a curved surface. The edges are maximally exposed. **b** and **c** Cathodic polarization curves of MoSe_2_ and WSe_2_ nanofilms on carbon fiber paper compared with those on mirror polished glassy carbon as well as a blank carbon fiber paper substrate [92], © American Chemical Society 2013.** d** STEM images of TiO_2_@MoS_2_ after exfoliation. **e** TEM images of TiO_2_@MoS_2_ after exfoliation. **f** H_2_ production rate over exfoliated TiO_2_@MoS_2_ at each hour of the HER [93] © Angew. Chem. 2017

The special structure of the curved substrate could provide a source of stress for the nanosheets grown on the surface that would cause strain, extension or extrusion. Cui et al. [92] prepared vertically aligned MoSe_2_ and WSe_2_ nanofilms on carbon fibers with 2D nanostructures (Fig. 7a). Vertically placed layers form strong bonds with the substrate and deformation occurs as the substrate bends, exposing more active sites. The catalytic activity of MoSe_2_ and WSe_2_ nanofilms on carbon fiber substrates was investigated in 0.5 M H_2_SO_4_ solution using a typical three-electrode electrochemical cell. As show in Fig. 7b, c, the overpotentials were about 250 and 300 mV at a current density of 10 mA cm^−2^, exhibiting better catalytic activity than that of planar substrates. In addition, our group [93] prepared vertically erected layer less/multilayer deformable MoS_2_ nanosheets on highly porous electrospun TiO_2_ nanofibers by using this method and exfoliated them to further enhance the catalytic activity (Fig. 7d). The TEM images in Fig. 7e indicated that exfoliated nanosheets on the curved substrate become non-continuous. As shown in Fig. 7f, MoS_2_ nanosheets grown on a curved substrate showed a higher H_2_ yield after stripping than before stripping. This proves that the way of growing flexible nanosheets on curved substrate can effectively promote HER properties.

Maximizing the catalytic activity of single-atom catalysts is the key for single-atom catalysts in industrial applications. Anchoring single atoms on the curved support exposes the active site significantly that the introduced tunable strain can effectively optimize the catalytic activity of single atoms and promote HER performance [94–100].

**Fig. 8:**
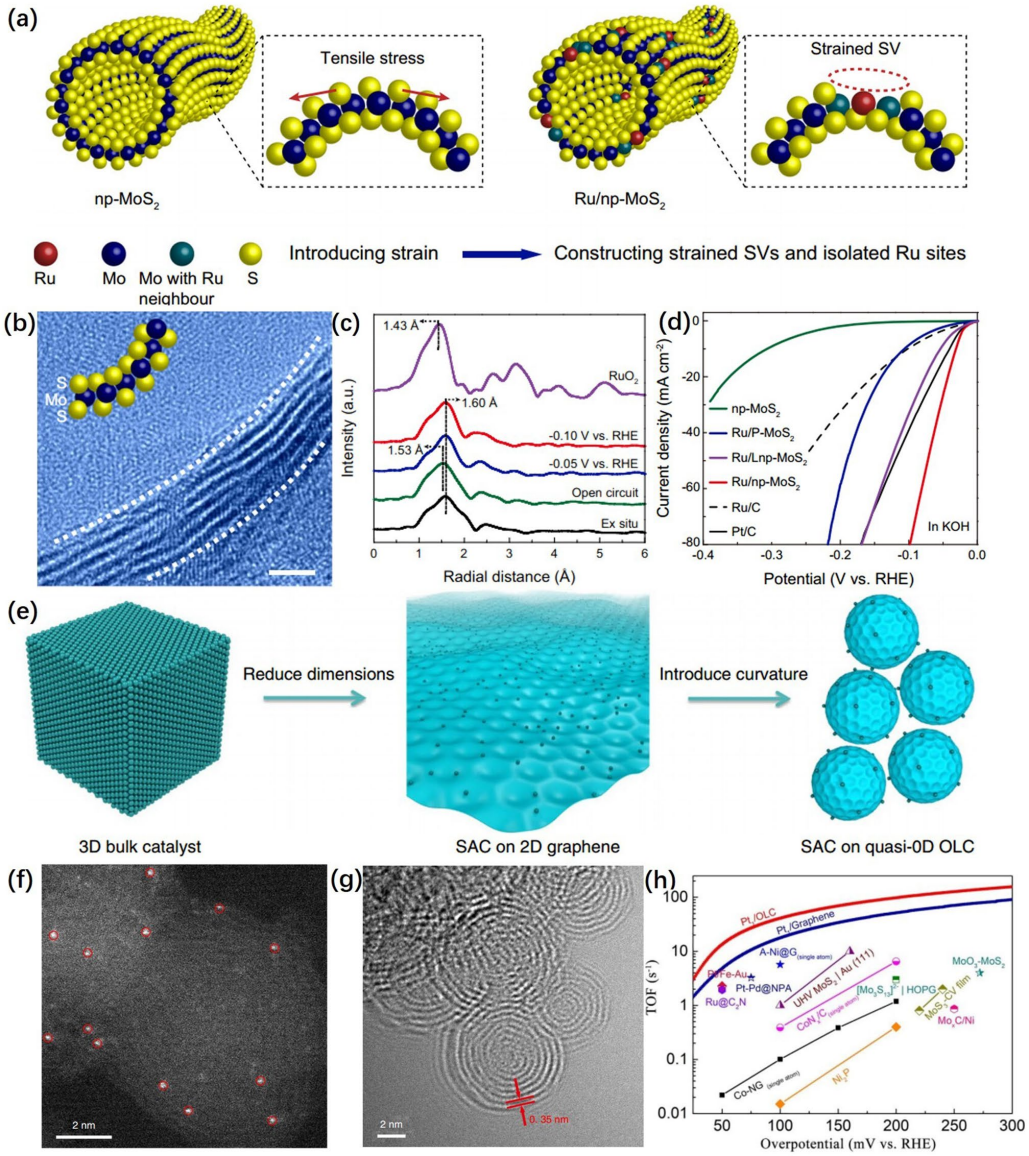
**a** Illustration of the construction Ru/np-MoS_2_. **b** HRTEM image of Ru/np-MoS_2_ from the cross-sectional view. **c** FT-EXAFS spectra of Ru/np-MoS_2_ recorded at different applied voltages. **d** ECSA-normalized polarization curves of Ru/np-MoS_2_, Ru/Lnp-MoS_2_, and Ru/P-MoS_2_ [101], © Springer Nature 2021. **e** The schematic illustrates the approach taken in this work, whereby catalytically active particles were reduced in size to a single-atom form and the dimensionality of the catalyst support was reduced by using quasi-0D OLCs. **f** HAADF-STEM image of Pt_1_/OLC clearly displays the Pt single atoms (highlighted by red circles) randomly dispersed on the OLC supports. **g** Transmission electron microscopy image of Pt_1_/OLC shows a multishell fullerene structure with a layer distance of 0.35 nm. **h** Turnover frequency curve of Pt_1_/OLC and Pt_1_/Graphene, in comparison with other previously reported catalysts [102], © Springer Nature 2019

Tan et al. [101] constructed MoS_2_-based Ru single-atom catalysts, in which bending strain-tunable sulfur vacancies (SV) around the single-atom Ru sites were explored to accelerate the alkaline HER. As shown in Fig. 8a, the synthesized bicontinuous structure of nanoporous MoS_2_ (np-MoS_2_) is composed of interconnected nanotubes with concave curvature and convex curvature. Isolated Ru atoms were introduced on the np-MoS_2_ substrate to establish SV. The HRTEM image in Fig. 8b showed the atomic-level bending of np-MoS_2_ and the atomic layer structure of multiple MoS_2_. Figure 8c shows the corresponding FT-EXAFS spectra of Ru/np-MoS_2_ at different applied potentials, confirming that the coordination environment of Ru atoms is distorted. As shown in Fig. 8d, the obtained Ru/np-MoS_2_ catalysts require an overpotential of 30 mV at a current density of 10 mA cm^−2^. The tip structure of curved catalysts could possess a local electric field, and the reactants would have a local high concentration distribution around the tip structure of the catalyst, thus accelerating the kinetic process of electrochemical reactions.

Song et al. [102] prepared high curvature onion-like nanospheres of carbon (OLC) loaded Pt single-atom catalysts (Pt_1_/OLC) based on nanodiamond (DND) (Fig. 8e). The HAADF-STEM image of Pt_1_/OLC in Fig. 8f showed that Pt on OLC carriers was in the single-atom state and no nanoparticles or clusters were formed. Meanwhile, Fig. 8g shows that a typical multishell fullerene structure was existed in the annealed DND. The turnover frequency (TOF) was used to quantify the catalytic efficiency of each Pt site. As shown in Fig. 8h, the TOF values of Pt SACs were significantly higher than those of most of HER catalysts under various overpotentials. This method of single atom catalyst supported by high curvature carrier successfully enhanced the activity of single atom site and improved HER performance by introducing controllable bending strain and regulating the electronic structure of single atom.

#### Flexible Deformable Substrate

Wrinkle engineering that utilizes flexural wrinkling or bending deformation is an effective way to change and tune the mechanical, physical and chemical properties of 2D crystals [103–107]. The flexible substrate can be used for generating micro-wrinkles of flexible materials due to its high ductility, high flexibility and strain controllability. The tight contact makes the flexible substrate transfer stress to the load well and regulates the bending degree of the flexible catalytic material to obtain the best catalytic performance [28, 108, 109].

**Fig. 9:**
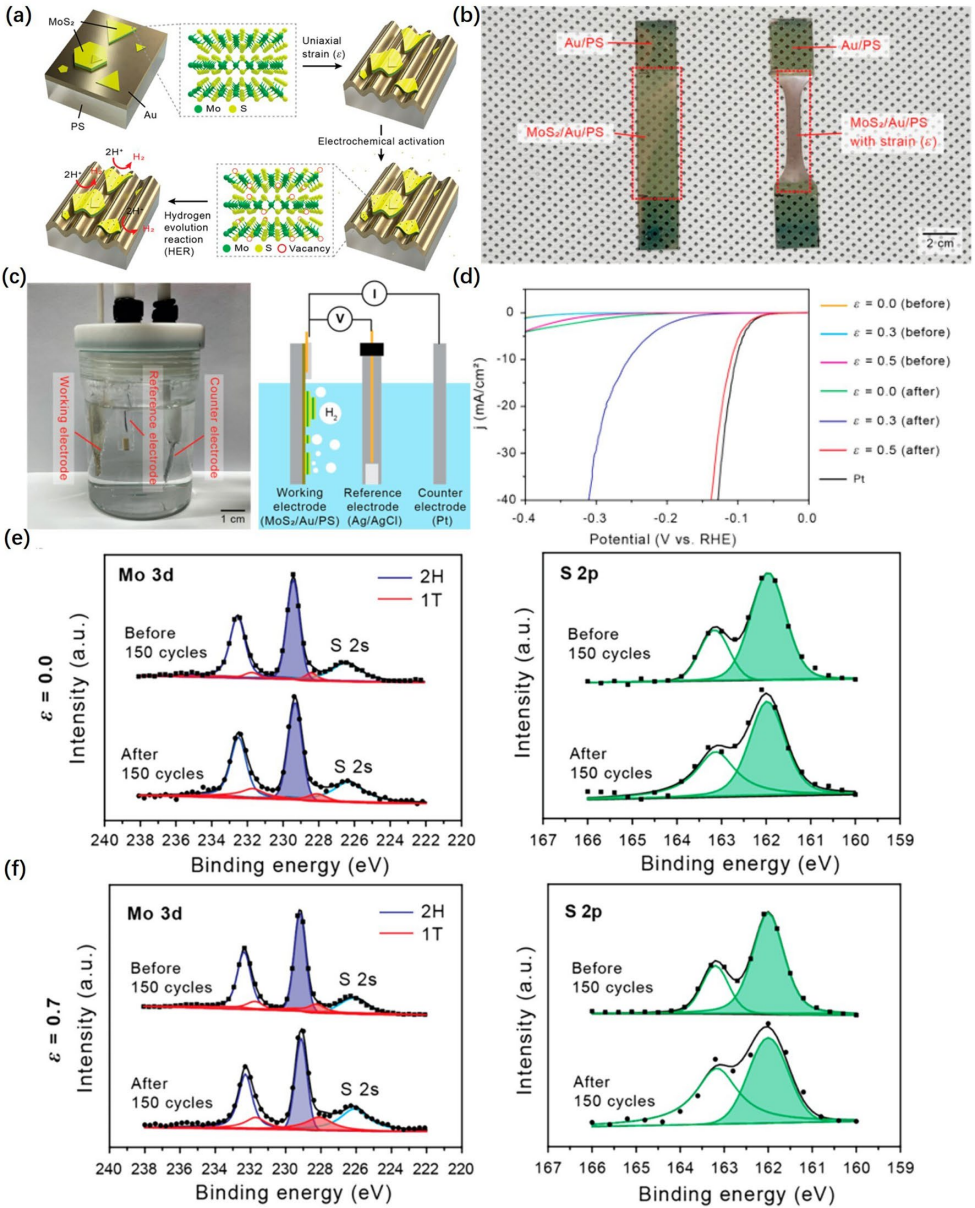
**a** Schematic illustration of micro-wrinkling and EC processes to prepare MoS_2_ HER catalysts. **b** Formation of wrinkled MoS_2_ and **c** electrochemical three-electrode system for HER, composed of a WE of MoS_2_/Au/PS, a reference electrode of Ag/AgCl, and a counter electrode of Pt. **d** iR-corrected polarization curves from the structured MoS_2_ with different strains before and after electrochemical activation (150 cycles of linear sweep voltammetry). XPS Mo 3*d* and S 2*p* peaks of the **e** pristine and **f** strained MoS_2_ before and after CV [110], © American Chemical Society 2022

The nanosheets with wrinkle structure by flexible substrate were fabricated from Lee et al. [110]. As shown in Fig. 9a, b, the MoS_2_ sheets were transferred onto gold films, which were deposited on pre-strained thermoplastic substrates. The macroscopic strain (*ε*) applied to the substrates was able to be adjusted by controlling the heating time. Based on the difference in E between the MoS_2_ flakes covered on the gold film and the substrate, wrinkles were formed entire sample surfaces. The structured MoS_2_ loaded on Au wrinkling as the working electrode (WE) was electrochemically (EC) activated by CV in a three-electrode system containing Pt foil (counter electrode, CE) and Ag/AgCl reference electrode (RE) (Fig. 9c). The HER performance of MoS_2_ was significantly improved after strain synergistic CV cycling. As illustrated in Fig. 9d, at high current density, the overpotential of MoS_2_ with *ε* 0.5 is 0.06 V, which is close to Pt. X-ray photoelectron spectroscopy (XPS) analysis verified the generation of S vacancy and the phase transition from 2H to 1T by CV, and the proportion of 1T peaks increased with increasing* ε* (Fig. 9e, f). This showed that the deformation of the catalyst could cause the phase transition and improve the catalytic activity.

**Fig. 10:**
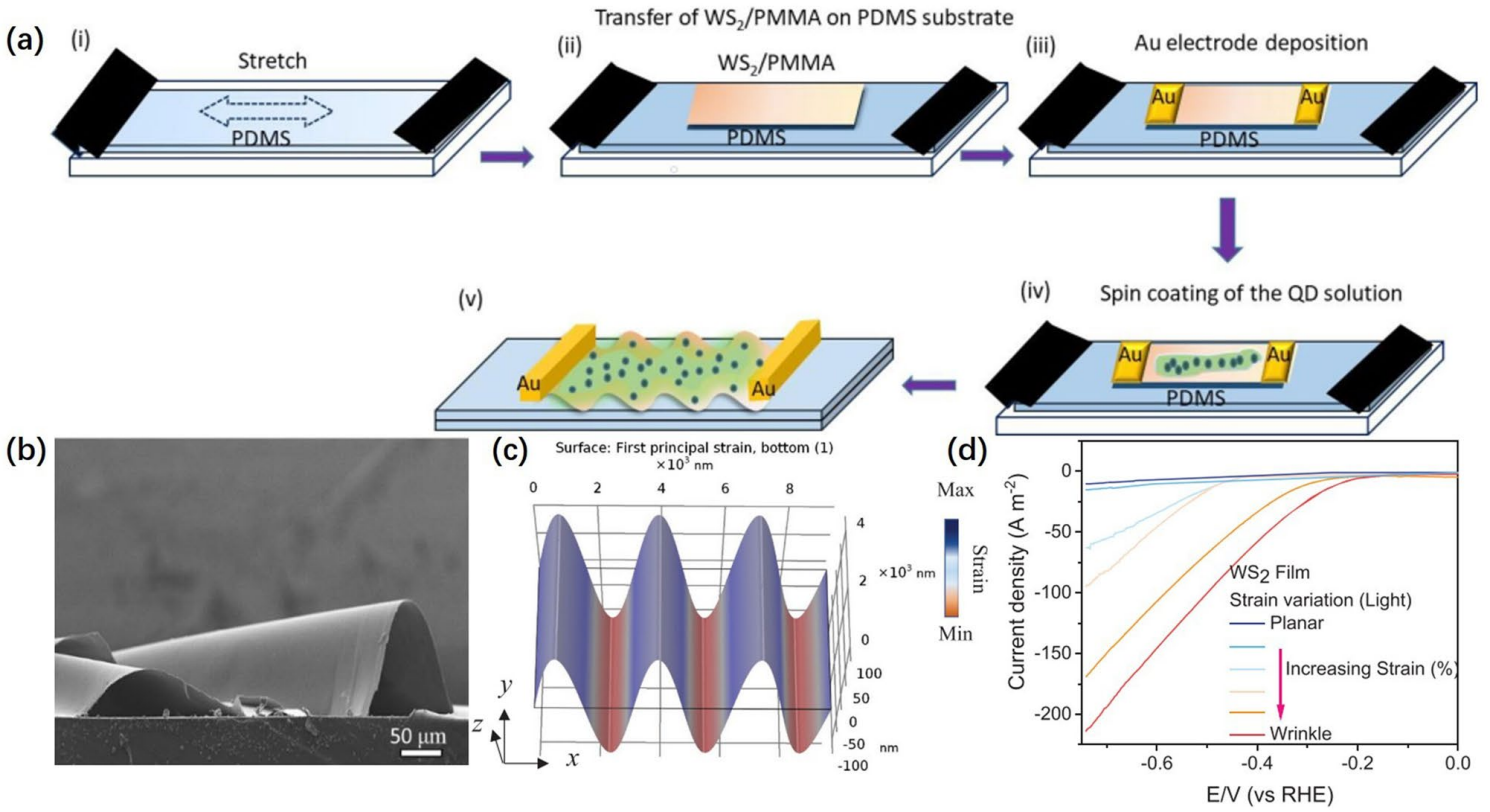
**a** Schematic diagram of micro-wrinkling fabrication in different steps. **b** SEM images of WS_2_ wrinkle structure. **c** Simulated strain distribution along wrinkle. **d** LSV curve of WS_2_ film with the increasing strain from planar to wrinkled structures [111], © John Wiley & Sons, Inc 2023

In addition, Chen et al. [111] prepared WS_2_ nanofilms with wrinkled structure by slacking the pre-strained substrate. As shown in Fig. 10a, when the pre-strained polydimethylsiloxane (PDMS) substrate relaxes, WS_2_ would have a large compressive strain to form wrinkles. The SEM image in Fig. 10b confirmed the obvious wrinkles of the WS_2_ monolayer film. This suspended structure could reduce the interaction with the substrate to enhance the charge carrier mobility. The simulated strain distribution through the perimeter of the fold is shown in Fig. 10c, indicating the presence of tensile strain in the fold structure. As shown in Fig. 10d, the onset potential has been decreased from − 0.38 to − 0.12 V vs RHE and the current value has been increased from ~ 3.5 to ~ 104.7 mA cm^−2^ at − 0.5 V vs RHE with the increasing strain. Micro-wrinkling induced by flexible substrate has abundant strain on the surface which can be accurately grasped by the regulation of the substrate. The strain could effectively improve the charge carrier mobility and optimize the reaction kinetics, thus enhancing HER activity.

### Deformation Based on Sacrificial Template

The flexible characteristics of two-dimensional materials give it a certain plasticity. Flexible nanomaterials can be molded into tubular structures by anodic aluminum oxide (AAO) templating to prepare catalysts with excellent catalytic properties [112–115].

**Fig. 11:**
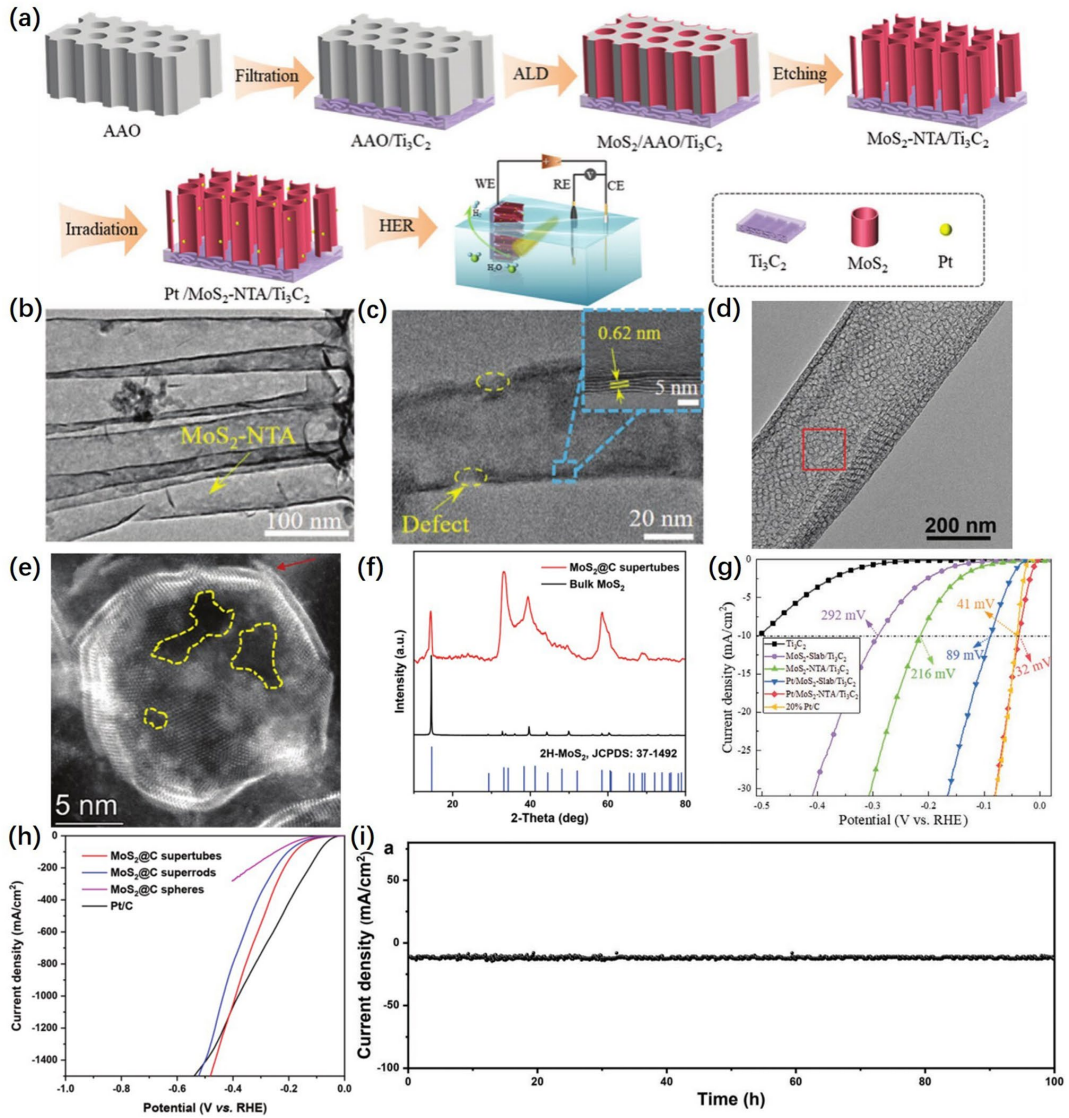
**a** Experimental flowchart for the synthesis processes of Pt/MoS_2_-NTA/Ti_3_C_2_. **b** TEM image of MoS_2_-NTA in MoS_2_-NTA/Ti_3_C_2_. **c** HRTEM image of the single MoS_2_ nanotube in Pt/MoS_2_-NTA/Ti_3_C_2_, the inset: partial enlargement displaying the MoS_2_ interlayer spacing [116], © Wiley-VCH GmbH 2022. **d** The SEM image of a single MoS_2_@C supertubes. **e** HAADF-STEM image of a single pore containing atomically curved MoS_2_. The arrow indicates the fractured MoS_2_ layers and the yellow contours indicate the voids at the basal plane of MoS_2_ [117], © Wiley-VCH GmbH 2023. **f** XRD patterns of MoS_2_@C supertubes and bulk 2H-MoS_2_ [117], © Wiley-VCH GmbH 2023. **g** HER polarization curves for commercial Pt/C and prepared samples in 0.5 m H_2_SO_4_ [116], © Wiley-VCH GmbH 2022. **h** Polarization curves at high current densities. **i** Durability tests of MoS_2_@C supertubes at 10 mA cm^−2^ [117], © Wiley-VCH GmbH 2023

Liu et al. [116] prepared titanium carbide (Ti_3_C_2_)-supported MoS_2_ nanotube arrays (MoS_2_-NTA) with controlled wall thickness and diameter by atomic layer deposition (ALD) technique based on anodic aluminum oxide (AAO) template sacrifice strategy (Fig. 11a). The TEM image in Fig. 11b showed the formation of MoS_2_-NTA on the substrate after NaOH etching of AAO. The HRTEM image of the single MoS_2_ nanotube in Fig. 11c showed a stripe spacing of 0.62 nm for Pt/MoS_2_NTA/Ti_3_C_2_ nanotubes, indicating that MoS_2_ grows layer by layer from single-walled nanotubes to multiwalled nanotubes with abundant defects. The graphene frameworks with tubular array structures based on AAO templates were fabricated from Li et al. [117]. The MoS_2_@C van der Waals supertubes were formed by restricting the epitaxial growth of several layers of bent MoS_2_ within the tubular mesoporous graphene framework.

The tubular graphene framework exists in the form of 1D arrays, with individual tubes being open and having ordered mesoporosity. As shown in Fig. 11d, the SEM image demonstrated the MoS_2_ layers within the mesopores on the nanotube. HAADF-STEM with aberration correction in Fig. 11e showed the bending nature of the MoS_2_ layer at the atomic scale. Meanwhile, the MoS_2_ layer tends to fracture near the corners of the mesopores (indicated by the arrows in Fig. 11e. In addition, a considerable number of structural defects, such as voids (indicated by the dashed outline in Fig. 11e. The X-ray diffraction (XRD) pattern of the MoS_2_@C supertube showed the characteristic peaks of 2H-MoS_2_, confirming the ultra-thin thickness of the MoS_2_ layer (Fig. 11f). The LSV curves of the electrocatalytic performance in Fig. 11g, h indicated that the nanotube prepared by AAO template method has excellent HER properties [118–120]. Figure 11i shows the stability tests performed on the catalyst of this structure, confirming that the catalyst of this structure could maintain HER performance for 100 h at 10 mA cm^−2^ without significant degradation. Therefore, the nanotubes prepared by the AAO template method can be used as a carrier for the preparation of various highly active HER catalysts effectively.

## HER Catalytic Mechanisms of Deformable Catalytic Materials

Exploring the catalytic mechanism of deformable catalytic materials is very important for the further design of highly active catalysts. From the view of HER mechanism, we summarized how deformable catalytic materials affect HER catalytic performance. The deformation of the catalytic material would introduce strain and vacancy, which could reduce the energy barrier of the Volmer step and lead to a faster Volmer step. Specifically, the deformable catalyst could optimize *ΔG*_H_^*^ under acidic conditions and reduce the energy barrier of water dissociation under alkaline conditions [121, 122]. In addition, the deformation of the catalyst optimizes electronic structure and improves electron transport efficiency, thus speeding up the Heyrovsky and Tafel steps.

**Fig. 12:**
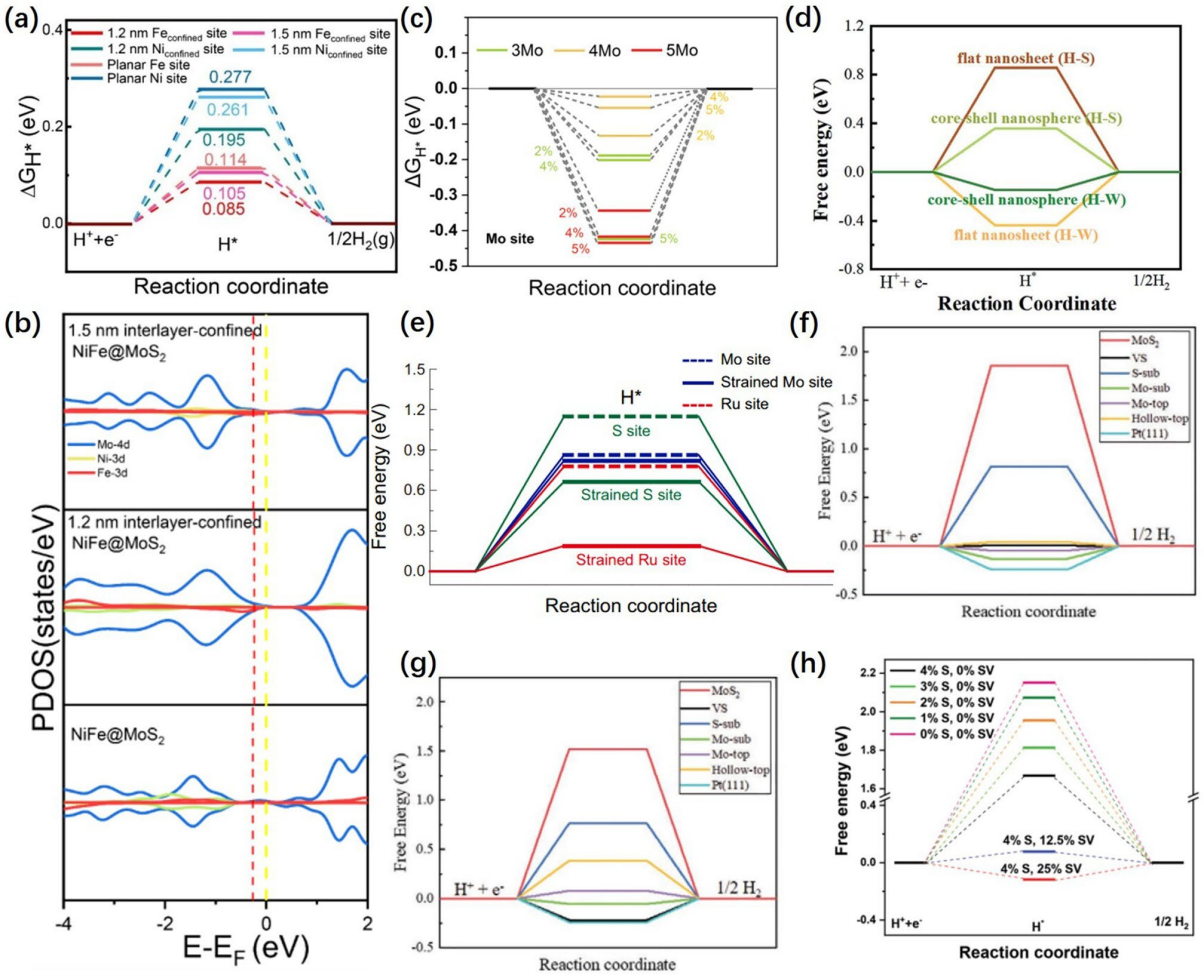
**a** Free energy diagram of HER process at Fe, Ni sites under interlayer confinement/plane condition. **b** PDOS results for 1.5 nm interlayer-confined NiFe@MoS_2_, 1.2 nm interlayer-confined NiFe@MoS_2_ and planar NiFe@MoS_2_ [72], © John Wiley & Sons, Inc 2023. **c** Calculated free-energy diagram of HER for pure MoS_2_ model under 2, 4, and 5% uniaxial/biaxial strain conditions with 5, 4, and 3 coordination number of Mo sites under biaxial strain [85], © Wiley-VCH GmbH 2022. **d** Free energy versus the reaction coordinate of HER at the basal planes of the flat WS_2_ nanosheet and W@WS_2_ CSNS [87], © The Royal Society of Chemistry 2021. **e** Free energy diagrams for hydrogen adsorption at S, Mo sits [101], © Springer Nature 2021. The *∆G*_H_^*^ versus the reaction coordinates of **f** MoS_2_-NT and **g** MoS_2_-slab [116], © Wiley-VCH GmbH 2022. **h** Free energy versus the reaction coordinate of HER for 2H-MoS_2_ with the S-vacancy (SV) and strain (S) [117], © Wiley-VCH GmbH 2023. * versus the reaction coordinates of **f** MoS_2_-NT and **g** MoS_2_-slab [116], © Wiley-VCH GmbH 2022. **h** Free energy versus the reaction coordinate of HER for 2H-MoS_2_ with the S-vacancy (SV) and strain (S) [117], © Wiley–VCH GmbH 2023

The discrete Fourier transform (DFT) could be used to further investigation of the relationship between the structure and performance of deformed catalysts. Yuan et al. [72] confirmed that the interlayer-confined nanoscrolls exhibited more favorable adsorption strengths and higher catalytic activity for acid water splitting in the constrained metal active center with the help of DFT (Fig. 12a). Projected state density (PDOS) indicated that the center of the d band of the interlayer confined NiFe@MoS_2_ nanoscroll catalyst was closer to the Fermi level (Fig. 12b), showing more favorable H_2_O adsorption, dissociation, and enhanced electron transport capacity. Fan et al. [85] calculated the* ∆G*_H_^*^ values of Mo sites with different coordination numbers constructed by introducing S vacancies under biaxial strain (Fig. 12c). Finally, it was confirmed that moderate biaxial strain and S vacancies enhanced the interaction between H intermediates and adsorption sites, accelerating the desorption of H from S vacancy MoS_2_ and improving the catalytic activity. Shen et al. [87] verified the contribution of tensile strain to catalyst activity through DFT calculations (Fig. 12d). By introducing tensile strain,* ∆G*_H_^*^ at S and W sites on W@WS_2_ CSNS were significantly decreased, and the catalytic activity was improved. Based on DFT calculation, Tan et al. [101] confirmed that tensile strain could reduce the Volmer-order energy barrier of single-atom Ru site, resulting in rapid Volmer-order change (Fig. 12e). Meanwhile, the application of strain could decrease *∆G*_H_^*^ for Ru and S sites to improve the ability of the H–H coupling.

Liu et al. [116] calculated the *∆G*_H_^*^ values for MoS_2_ with planar structure (MoS_2_-slab) and nanotube structure (MoS_2_-NT) (Fig. 12f, g). The S-sub and Mo-sub of MoS_2_-NT had lower *∆G*_H_^*^ values than that of MoS_2_-slab, which confirmed that the high curvature of the surface of the nanotubes enhances the H adsorption strength at the active site and improves the catalytic activity. Li et al. [117] confirmed by DFT calculation that the strain of MoS_2_ layer curvature and S-vacancy can reduce *∆G*_H_^*^ to increase HER catalytic activity (Fig. 12h). The DFT calculation is a bridge linking the structure–activity relationship between deformation and catalytic activity of catalytic materials. Advanced in situ characterization could be used to further investigate the mechanism in the future.

## Conclusion and Outlook

Flexible micro–nanostructures are undoubtedly evolving from monotonic to diversified. In order to fully understand the relationship between deformation and catalytic performance, it is necessary to have new insights into the influence of flexible structure on the reactivity, electronic structure and reaction kinetics of catalytic reactions. This review summarizes the deformation of flexible materials in the catalytic process and the design of excellent catalysts based on the mechanical flexibility of low-dimensional nanomaterials. We believe that the influences of deformation on flexible catalysts mainly have the following aspects: (1) The low-dimensional nanomaterials with nanoscale curved geometries incur the change of electronic structure of catalyst, which could accelerate the electron transfer in the electrocatalytic process. (2) The mechanical deformation would trigger new favorable structures such as vacancies or phase transitions to improve catalytic activity. (3) The unique mechanical properties of flexible materials are beneficial for the higher mass transfer efficiency and the faster reaction kinetics.

The application of deformable catalysts has attracted extensive attention. It has been reported that the HER activity of thin film palladium electrocatalysts can be improved by applying tensile strain to flexible working electrodes. In addition to its application in the HER, deformable catalytic materials have potential uses in various other chemical reactions and energy conversion systems. Qiao et al. [123] reported three-dimensional nanosheet superstructure (NF-MOF_5 h_) by stacking metal organic framework (MOF) nanosheets on the surface of nickel foam (NF). The stacked three-dimensional nanosheets superstructure accelerated reaction kinetics and electron transport efficiency thus optimizing OER performance. Yang et al. [124] prepared a helical carbon structure with abundant high-curvature surface which was realized by carbonization of helical polypyrrole that was templated from self-assembled chiral surfactants for improving the electrocatalytic activity of oxygen reduction reaction (ORR). Chen et al. [125] achieved efficient NRR catalysis by anchoring single-atom Au onto a bicontinent nanopore MoSe_2_ (np-MoSe_2_). It is worth noting that in addition to transition metal dichalcogenides (TMDs) other low-dimensional materials, such as carbon-based materials, MXene and metallenes are also deformable materials [126–128]. Therefore, further research on the deformation catalyst is still necessary. Great progress has been made in the design of deformation catalysts with artificial intelligence (AI) assistance. By establishing the model and algorithm, the deformation behavior of the material can be simulated and the morphology structure can be optimized. This can help design highly active catalysts with expected deformation and mechanical properties. Therefore, the combination of machine learning and computational chemistry methods can effectively accelerate the development process of catalysts [129, 130]. In fact, molecular dynamics (MD) simulations play a key role in the study of the structural morphology of catalysts, which can simulate the morphology structure, mechanical properties and calculate the electronic structure of materials at the nanoscale. Compared with the experimental approach, MD can not only effectively reduce the cost of research, but also provide richer information about materials. In addition, the study of catalytic mechanisms in the HER process is essential for the rational design of catalysts and efficient adaptive electrocatalytic processes. This requires advanced in situ characterization of catalysts with unique deformed structures. For example, based on the use of in situ Raman spectroscopy, in situ XRD, in situ FT-IR and in situ HRTEM, we can gain a clearer understanding of the catalyst structure evolution, electron transfer process and the adsorption/desorption process to uncover the real active center.

In conclusion, although deformable catalytic materials have been widely studied and applied, several challenges still remain. Researchers from different fields are trying to solve these challenges through communication and collaboration. We believe that deformable catalytic material with excellent flexible structure would give a strong impetus to the development of novel catalysts. It provides a strategic choice for the rational design of low-cost and high-performance industrialized electrocatalysts.
